# Aldolase is a sensor for both low and high glucose, linking to AMPK and mTORC1

**DOI:** 10.1038/s41422-020-00456-8

**Published:** 2020-12-21

**Authors:** Mengqi Li, Chen-Song Zhang, Jin-Wei Feng, Xiaoyan Wei, Cixiong Zhang, Changchuan Xie, Yaying Wu, Simon A. Hawley, Abdelmadjid Atrih, Douglas J. Lamont, Zhichao Wang, Hai-Long Piao, D. Grahame Hardie, Sheng-Cai Lin

**Affiliations:** 1 State Key Laboratory for Cellular Stress Biology, School of Life Sciences, Xiamen University, Xiamen, Fujian 361102 China; 2 Division of Cell Signalling and Immunology, College of Life Sciences, University of Dundee, DD1 5EH Dundee, Scotland UK; 3 Fingerprints Proteomics Facility, School of Life Sciences, University of Dundee, DD1 5EH Dundee, Scotland UK; 4 Department of Molecular & Cellular Endocrinology, Diabetes and Metabolism Research Institute, City of Hope Medical Center, Duarte, CA 91010 USA; 5 CAS Key Laboratory of Separation Science for Analytical Chemistry, Dalian Institute of Chemical Physics, Chinese Academy of Sciences, Dalian, Liaoning 116023 China

**Keywords:** Nutrient signalling, Lysosomes, TOR signalling

Dear Editor,

As well as glucose being the major carbon nutrient for most cells, its availability also acts as a gate-keeper exerting a switch between anabolic and catabolic metabolism, with the protein kinases mTORC1 and AMP-activated protein kinase (AMPK) being the two master controllers.^1^ In low glucose, AMPK is activated and phosphorylates a wide range of downstream targets to maintain energy homeostasis, by switching on catabolic pathways while switching off ATP-consuming processes.^1^ In high glucose, mTORC1 is activated and shifts the metabolic program of the cell towards anabolic metabolism.^1^ It is already known that low glucose, and hence low levels of the glycolytic intermediate fructose-1,6-bisphosphate (FBP), can be sensed by aldolase to trigger AMPK activation via the lysosomal pathway.^2,3^ This occurs independently of increases in AMP and ADP, the classical activators of AMPK.^2^ Although it is clear that mTORC1 is also regulated by glucose availability at multiple levels, the underlying mechanisms have not been fully elucidated.

To maintain its activity, mTORC1 has to be located on the surface of the lysosome, where it is activated by binding to two classes of lysosome-localized small G proteins, i.e., Rheb and Rags. It has been shown that the mTORC1:Rheb interaction is regulated by GAPDH: when unoccupied by glyceraldehyde-3-phosphate (G3P), a glycolytic metabolite downstream of FBP, GAPDH inhibits the ability of Rheb to activate mTORC1.^4^ Rags are regulated by the Ragulator complex, which is associated with the vacuolar ATPase (v-ATPase), a multisubunit proton pump that hydrolyses ATP to provide the energy to acidify the lumen of the lysosome.^5^ In high glucose, the Ragulator converts RagA or RagB to their active GTP-bound forms, triggering translocation of mTORC1 to the lysosome.^6^ In low glucose, v-ATPase activity is inhibited, which in turn inhibits the Ragulator.^6^ It is important to note that an active v-ATPase is required to maintain the Ragulator activity, thus allowing the Rags to activate mTORC1.^5^ However, how glucose is sensed and relayed to the RAGs for mTORC1 activation has remained elusive.

We have previously shown that the lack of occupancy of aldolase by FBP in low glucose triggers AMPK activation, which prompted us to examine whether aldolase is also involved in the activation of mTORC1 in high glucose. However, knocking down all aldolases (ALDOA/ALDOB/ALDOC) in MEFs directly led to a strong inhibition of the v-ATPase, as evidenced by a decreased signal from LysoSensor Green DND-189 dye (Supplementary information, Fig. S1a), indicating that lysosomal pH was raised. This is consistent with previous findings that aldolase, as well as being a glycolytic enzyme, is also an integral component of the v-ATPase complex that is required for activity of the latter.^7^ The intrinsic requirement of aldolase for the integrity of the v-ATPase therefore precludes the use of ALDO knockdown or knockout approaches to study regulation of mTORC1 by aldolase. We utilized instead the D34S mutant of ALDOA, a mutation that does not significantly affect initial Schiff base formation between FBP and K230 of aldolase, but does block the carbon–carbon cleavage that converts FBP to DHAP and G3P, which is mediated by D34^8^ (Fig. 1a). This mutant therefore still binds FBP even in low glucose, so that its expression mimics a high glucose state. We indeed found that AMPK activation,^2^ as well as v-ATPase inhibition (Supplementary information, Fig. S1b) was blocked in these cells, regardless of the presence or absence of glucose in the medium. By contrast, the K230A mutant fails to form a Schiff base with FBP, thus preventing FBP binding.^8^ Expression of this mutant therefore mimics a low glucose state and leads to constitutive activation of AMPK.^2^ Expression of the D34S mutant in MEFs maintained the activity of mTORC1 even in low glucose, while expression of K230A caused inhibition of mTORC1 even in high glucose (Fig. 1b); similar results were obtained in HEK293T cells (Fig. 1c).

**Fig. 1:**
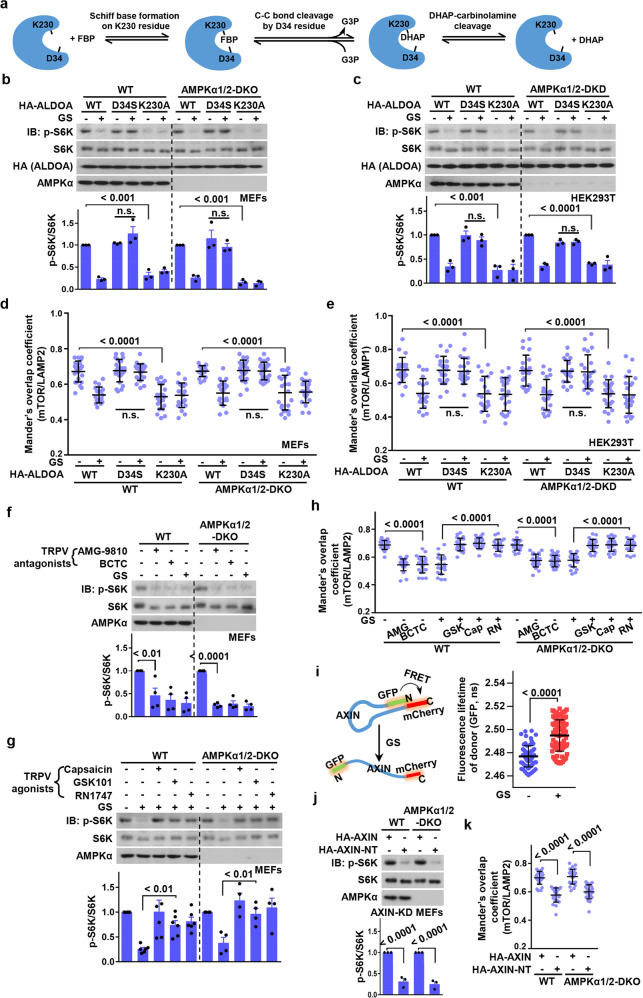
Aldolase is a sensor of glucose availability for mTORC1 regulation. **a** Schematic diagram of catalytic mechanisms of aldolase.^8^ **b–e** Expression of the D34S aldolase mutant retains mTORC1 activity in low glucose, while the K230A mutant inhibits mTORC1 even in high glucose. Wild-type and AMPKα1/2-DKO MEFs (**b**, **d**), and wild-type and AMPKα1/2-DKD HEK293T cells (**c**, **e**) were infected with lentivirus expressing HA-tagged ALDOA, the D34S or K230A mutants; the cells were then incubated in DMEM medium with 8 mM glucose, or were starved for glucose (GS) for 2 h. mTORC1 activity was assessed by determining of p-S6K and S6K levels by immunoblotting, followed by densitometry analysis (**b**, **c**, data are means ± SEM, *n* = 3, with *P* values calculated by ANOVA). The lysosomal localization of mTOR (**d**, **e**) was determined by immunofluorescent staining. mTOR, and the lysosomal marker LAMP2 (**d**) or LAMP1 (**e**) were stained, and Mander’s overlap coefficients were plotted as means ± SD, *n* = 19–24, with *P* values calculated by ANOVA (see representative images in Supplementary information, Fig. S2a, b). **f–h** Inhibition of TRPV impairs mTORC1 activity in high glucose, and activation of TRPV restores the mTORC1 activity in low glucose. Wild-type and AMPKα-DKO MEFs were treated with 5 μM AMG-9810 (AMG) or 10 μM BCTC for 30 min in DMEM medium containing 8 mM glucose (**f**, **h**), or glucose-starved for 2 h, followed by addition of 50 nM GSK101 (GSK), 100 nM capsaicin (Cap), or 0.7 μM RN1747 (RN) for 15 min (**g**, **h**). Levels of p-S6K and S6K were then determined by immunoblotting, followed by densitometry analysis (**f**, **g**, data are means ± SEM, *n* = 4–6; *P* values were calculated by ANOVA). The localization of mTOR (**h**) was determined by immunofluorescent staining as in (**d**), and Mander’s overlap coefficients were plotted as means ± SD, *n* = 20–26, with *P* values calculated by ANOVA (See representative images in Supplementary information, Fig. S3a). **i** The conformation of AXIN, determined by FRET–FLIM, changes in response to low glucose. MEFs were infected with lentivirus expressing AXIN1 tagged at the N-terminus with GFP (FRET donor) and at the C-terminus with mCherry (FRET acceptor). The resultant AXIN1 construct was named the M2M4 mutant, which carries mutations in the DIX domain to prevent intermolecular interactions,^15^ so that the FRET signals only reflect intramolecular interactions. Cells were incubated in DMEM medium with 8 mM glucose, or starved for glucose (GS) for 2 h, and the fluorescent lifetimes of GFP were measured and plotted as means ± SD; *n* = 70 and 105 for cells incubated in 8 mM and 0 mM glucose, respectively. *P* values were calculated by Student’s *t*-test. **j**, **k** Expression of AXIN-NT inhibits mTORC1 activity in high glucose. Wild-type and AMPKα-DKO MEFs with AXIN knockdown were infected with lentiviruses expressing HA-tagged AXIN and AXIN-NT. Cells were incubated in DMEM medium with 8 mM glucose. Levels of p-S6K and S6K were then determined by immunoblotting, followed by densitometry analysis (**j**, data are means ± SEM, *n* = 3; *P* values were calculated by Student’s *t*-test). The localization of mTOR (**k**) was determined by immunofluorescent staining as in (**d**), and Mander’s overlap coefficients were plotted as means ± SD, *n* = 22–26 with *P* values by ANOVA (see representative images in Supplementary information, Fig. 3b). All experiments in this figure were performed at least twice.

Note that activated AMPK can inhibit mTORC1 by directly phosphorylating its Raptor subunit, or TSC2, an upstream inhibitor of Rheb, or ULK1 to inhibit Rags.^1,9^ Importantly, to exclude the influence of AMPK, we also used AMPK-α1/2 DKO (double-knockout) MEFs and AMPK-α1/2 DKD (double-knockdown) HEK293T cells; the results confirmed that FBP-binding by aldolase is able to control mTORC1 independently of AMPK (Fig. 1b, c). We next examined whether the lysosomal localization of mTORC1 could be regulated by aldolase, and found that expression of the D34S aldolase mutant retained the lysosomal localization of mTORC1 in low glucose, while expression of the K230A mutant caused dissociation from the lysosome even in high glucose (Fig. 1d, e; Supplementary information, Fig. S2a, b). Similar results were obtained in AMPK-α DKO/DKD cells (Fig. 1d, e; Supplementary information, Fig. S2a, b). We have previously shown that the shortage of glucose (or more precisely FBP, after sensing by aldolase) is relayed to the v-ATPase by inhibiting TRPV channels acting upstream of it.^10^ Inhibition of TRPVs would also prevent high glucose from maintaining mTORC1 activity. Indeed, TRPV inhibitors AMG-9810 and BCTC, mimicking a low glucose state, impaired the activity as well as the lysosomal localization of mTORC1 in high glucose, in both WT and AMPKα1/2-DKO cells (Fig. 1f, h; Supplementary information, Fig. S3a). Conversely, activation of TRPV channels by agonists such as capsaicin (for TRPV1) or GSK101 and RN1747 (for TRPV4) restored the activation of mTORC1 in low glucose (Fig. 1g, h; Supplementary information, Fig. S3a). In conclusion, we propose that aldolase acts as a sensor for high glucose signalling to mTORC1.

It has been suggested that inactivation of v-ATPase by inactive TRPV channels in low glucose leads to conformational changes of the v-ATPase-Ragulator complex, providing a platform for AXIN docking, where AXIN in turns undergoes conformational changes for interacting with lysosomally localized AMPK.^3,10^ The conformational change of AXIN, an intrinsically disordered scaffold protein, is supported by the results from the FRET–FLIM experiment (Fig. 1i), which showed an increased fluorescence lifetime of the donor (GFP fused to the N-terminus of AXIN), as a result of decreased FRET between N- and C-termini of AXIN. Indeed, N-terminally truncated AXIN-NT, which lacks intramolecular autoinhibition provided by the C-terminal domain, exhibits higher affinity towards the v-ATPase and Ragulator than full-length AXIN.^3^ Interestingly, the autonomous binding of AXIN-NT to the Ragulator–v-ATPase complex alone is sufficient to inhibit mTORC1 (Fig. 1j, k; Supplementary information, Fig. S3b), further indicating that the lysosomal translocation of AXIN is a vital part of the mechanism of mTORC1 inhibition in low glucose.^3^

As well as mTORC1 regulation by aldolase via sensing of fluctuations in FBP (which decreases after glucose starvation by up to 10-fold in MEFs and 5-fold in HEK293T cells, Supplementary information, Fig. S4a, b; note that FBP, particularly in HEK293T cells, should be determined by capillary electrophoresis-mass spectrometry (CE-MS), rather than high-performance liquid chromatography-mass spectrometry (HPLC-MS) using pHILIC columns for it fails to separate FBP from its isomers, see Supplementary information, Fig. S4b–d), glucose may also be sensed by other mechanisms to modulate mTORC1. As shown above, expression of the ALDOA-D34S mutant maintains the activity of mTORC1 even in low glucose, when GAPDH would be unoccupied by G3P.^4^ This indicates that the Rag pathway for lysosomal anchoring of mTORC1 cannot be over-ridden by unoccupied GAPDH interfering with the interaction between mTORC1 and Rheb. This also confirms that Rags play a dominant role in sensing nutrient availability for activating mTORC1 as reported previously.^11^ However, the Rheb branch may play an augmentative role to maintain the activity of mTORC1, particularly when the Rag branch is disabled; in fact, Rheb has already been shown to play a dominant role in the absence of the Rag pathway.^12^ Similarly, exogenously supplied DHAP supports mTORC1 activation in cells lacking aldolase,^13^ possibly by binding to GAPDH after conversion to G3P. Since aldolase possesses bidirectional activity (Fig. 1a), the ability of the ALDOA-D34S mutant to maintain mTORC1 activity might also be attributed to its ability to bind DHAP in low glucose, although we previously attributed this to binding of FBP only. Evidence in support of this is that the K230 residue of the ALDOA-D34S protein aldolase from starved cells could be detected in both a phosphoglucitolylated (six-carbon, likely derived from FBP) and a phosphoglycerolylated (three-carbon from DHAP) form by mass spectrometry (Supplementary information, Fig. S4e). In addition, binding of DHAP or FBP leads to similar structural changes in the C-terminal tail of aldolase, which is required for the binding of TRPV.^10,14^ Therefore, aldolase may also act as a sensor for DHAP to regulate mTORC1. Remarkably, several other glycolytic enzymes and their substrates/products also participate in regulating mTORC1 in response to glucose availability at multiple sites (Supplementary information, Fig. S5).

## Supplementary information

Supplementary information

## References

[1] Gonzalez A, Hall MN, Lin SC, Hardie DG (2020). Cell Metab..

[2] Zhang CS (2017). Nature.

[3] Zhang CS (2014). Cell Metab..

[4] Lee MN (2009). Mol. Cell. Biol..

[5] Zoncu R (2011). Science.

[6] Efeyan A (2013). Nature.

[7] Forgac M (2007). Nat. Rev. Mol. Cell Biol..

[8] Morris AJ, Tolan DR (1993). J. Biol. Chem..

[9] Yoon I (2020). Science.

[10] Li M (2019). Cell Metab..

[11] Sancak Y (2008). Science.

[12] Demetriades C, Doumpas N, Teleman AA (2014). Cell.

[13] Orozco JM (2020). Nat. Metab..

[14] Heron PW, Sygusch J (2017). J. Biol. Chem..

[15] Schwarz-Romond T (2007). Nat. Struct. Mol. Biol..

