# Supplementary material for: Aldolase is a sensor for both low and high glucose, linking to AMPK and mTORC1
Source: Cell Res. 2020 Dec 21;31(4):478–81. doi: 10.1038/s41422-020-00456-8 (PMC8115481; doi:10.1038/s41422-020-00456-8)
Supplement: Supplementary file 1 — Supplementary information [file 41422_2020_456_MOESM1_ESM.pdf]

Supplementary information, Fig. S1

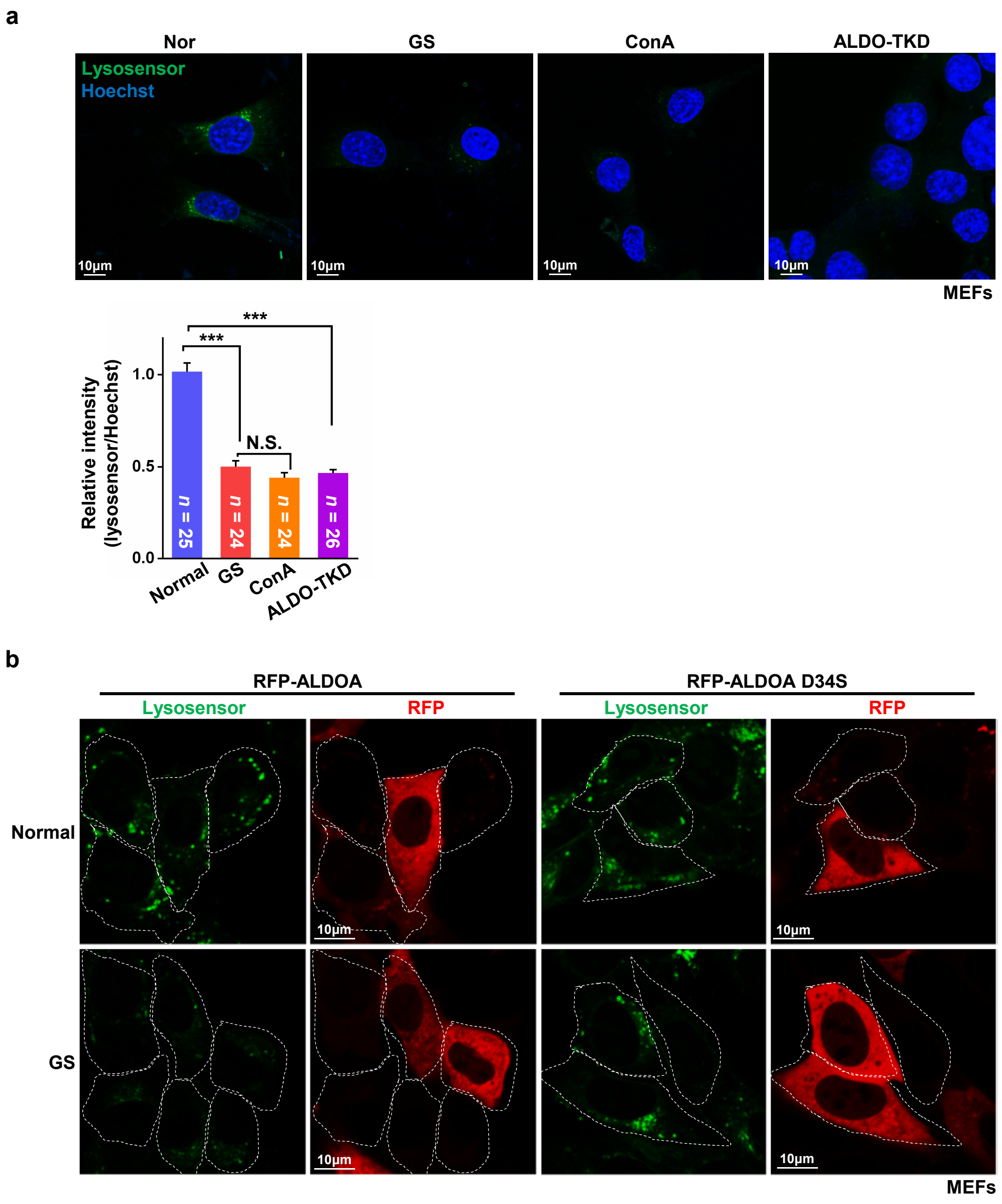

Supplementary information, Fig. S1 The activity of v-ATPase is inhibited in low glucose/FBP.

a The activity of v-ATPase, monitored as the acidity of lysosomes, is inhibited upon glucose starvation or aldolase knockdown. Wildtype MEFs, or MEFs with aldolase knocked down (ALDO-TKD), pre-loaded with LysoSensor Green DND-189 (green) and Hoechst (blue) dyes were cultured in DMEM with 8 mM glucose (Nor) or DMEM without glucose (GS) for 2 h, or treated with the v-ATPase inhibitor concanamycin A (ConA, 5 µM) as a control. The relative fluorescence intensities of LysoSensor (normalised to the intensity of Hoechst dye) were analysed, and representative images are shown. Results are plotted in the lower panel as mean ± s.e.m., and *P* values calculated by ANOVA are shown.

b FBP-bound to aldolase maintains the acidity of lysosome upon glucose starvation. MEFs stably expressing RFP-tagged ALDOA-D34S (red channel, right panel), or wild type ALDOA (red channel, left panel) as a control, were cultured in 8 mM glucose or were glucose-starved for 2 h. The pH of lysosomes was then determined by the fluorescence intensity of LysoSensor (green channel).

Experiments in this figure were performed at least twice.

Supplementary information, Fig. S2

a

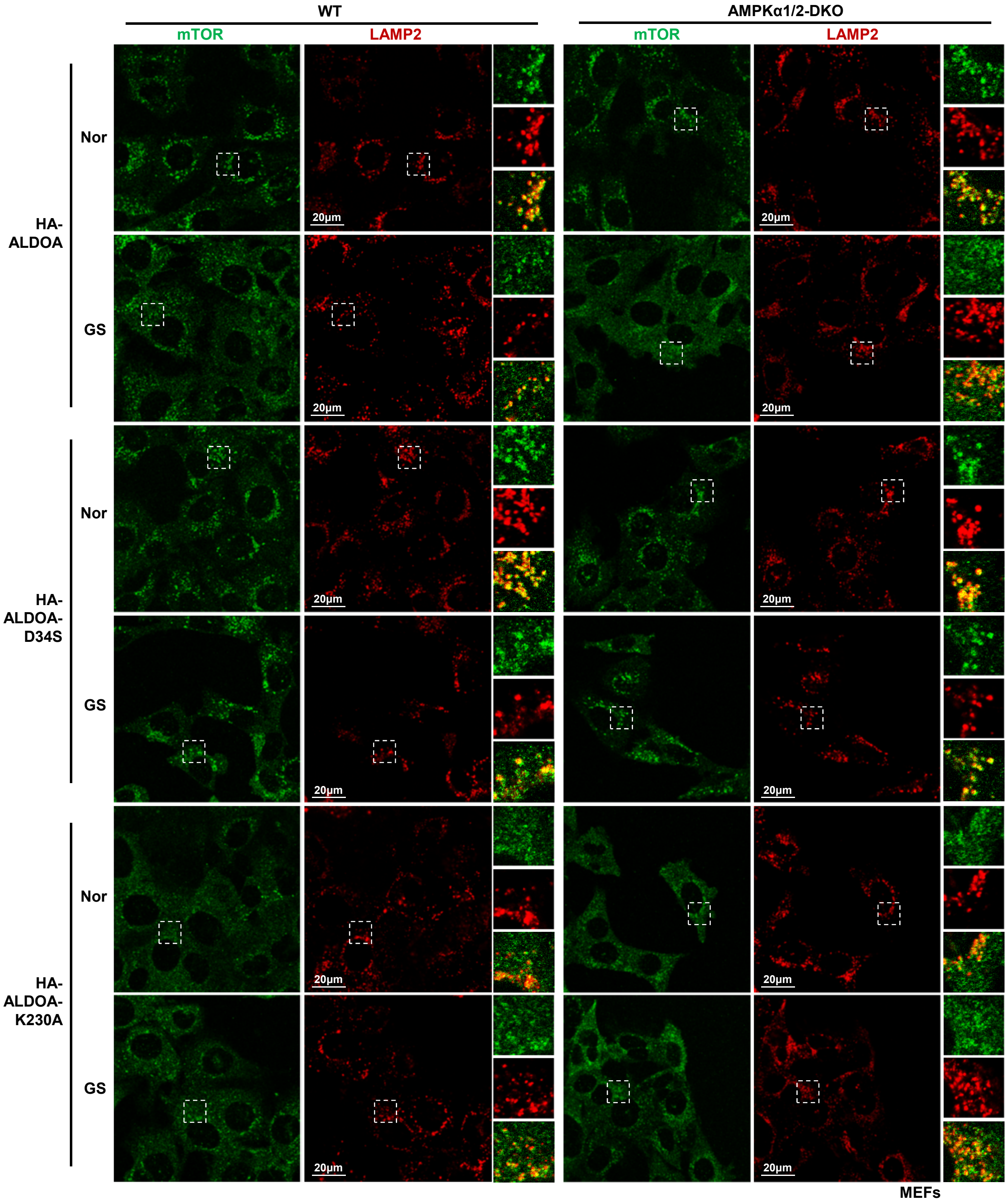

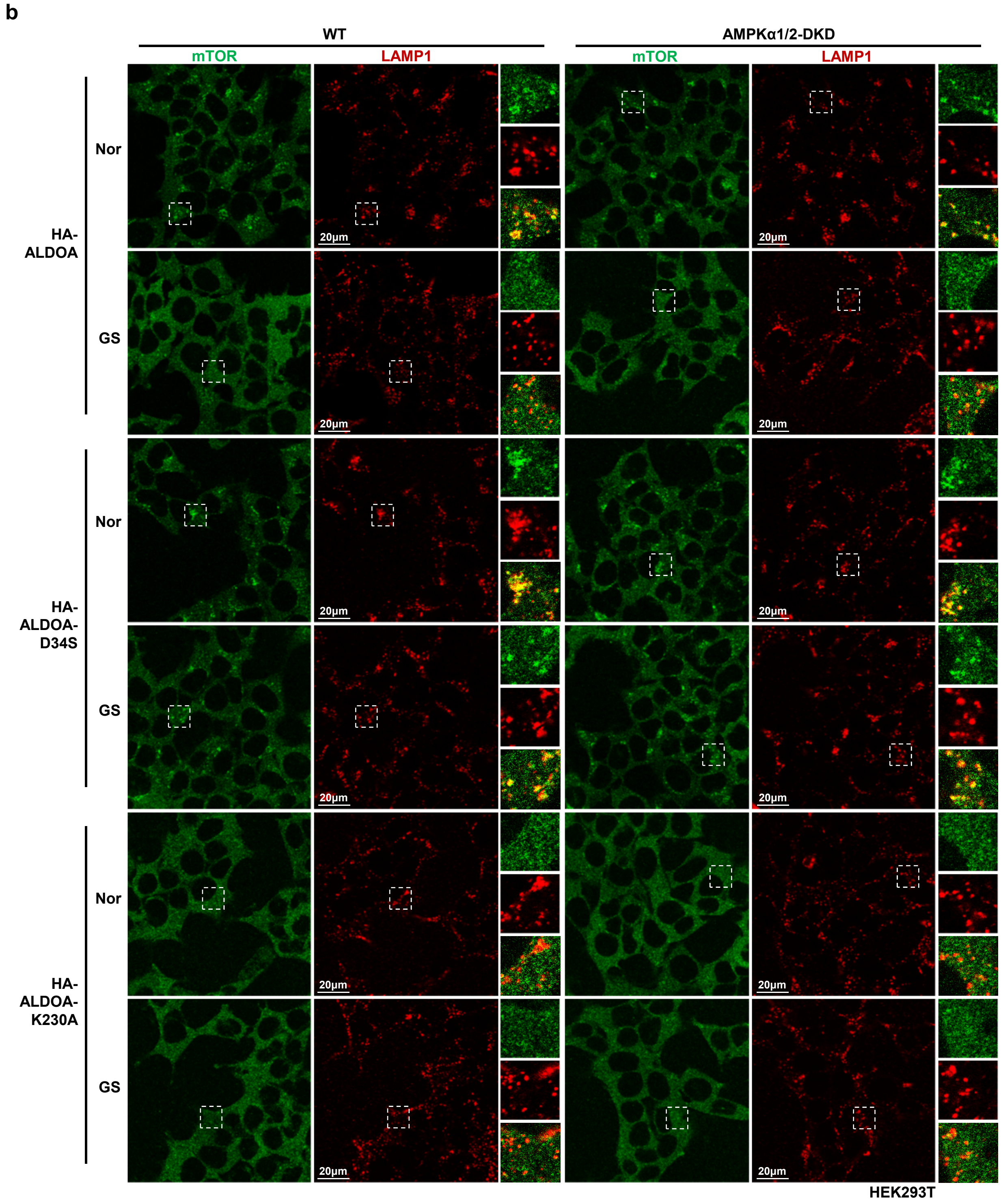

Supplementary information, Fig. S2 The substrate-binding state of aldolase is required for the regulation of mTORC1 localisation in response to glucose availability.

Wild-type and AMPKα-DKO MEFs (**a**) or AMPKα-DKD HEK293T cells (**b**) expressing wild-type ALDOA, the D34S or K230A mutants were incubated in DMEM with 8 mM glucose (Nor), or starved for glucose (GS) for 2 h. mTOR and the lysosome marker LAMP2 (**a**) or LAMP1 (**b**) were immunostained. Representative images are shown, and the areas defined by dashed boxes are enlarged as insets on the right. After superimposition of the red and green images, the yellow colour indicates overlap of the two proteins. See statistical analysis in Fig. 1d and 1e, respectively.

Experiments in this figure were performed at least twice.

Supplementary information, Fig. S3

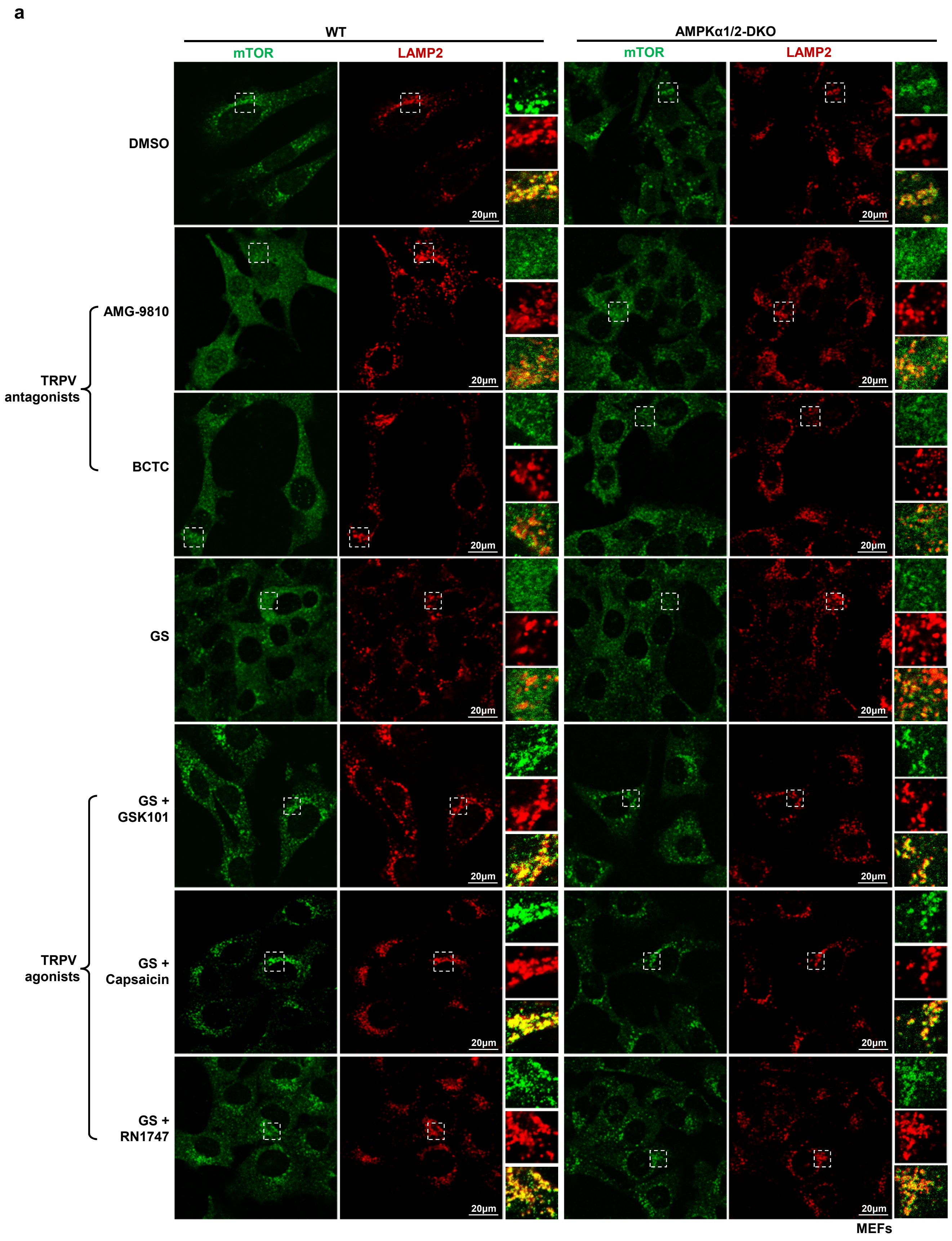

b

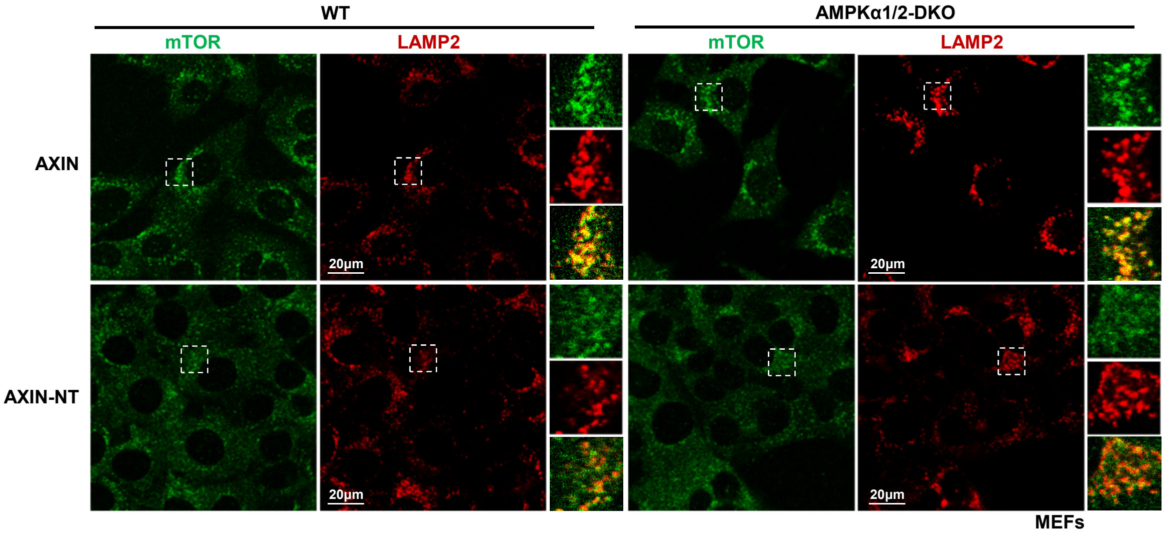

Supplementary information, Fig. S3 TRPV and AXIN regulate lysosomal localisation of mTORC1.

a Wild-type and AMPKα-DKO MEFs were treated with 5 µM AMG-9810 or 10 µM BCTC for 30 min in DMEM medium containing 8 mM glucose, or glucose starved for 2 h, followed by addition of 50 nM GSK101, 100 nM capsaicin, or 0.7 µM RN1747 for another 15 min. mTOR and LAMP2 were immunostained and representative images are shown. See statistical analysis in Fig. 1h.

b Wild-type and AMPKα-DKO MEFs with AXIN knockdown were infected with lentiviruses expressing HA-tagged AXIN and AXIN-NT. mTOR and LAMP2 were stained and representative images are shown. See statistical analysis in Fig. 1k.

Experiments in this figure were performed at least twice.

Supplementary information, Fig. S4

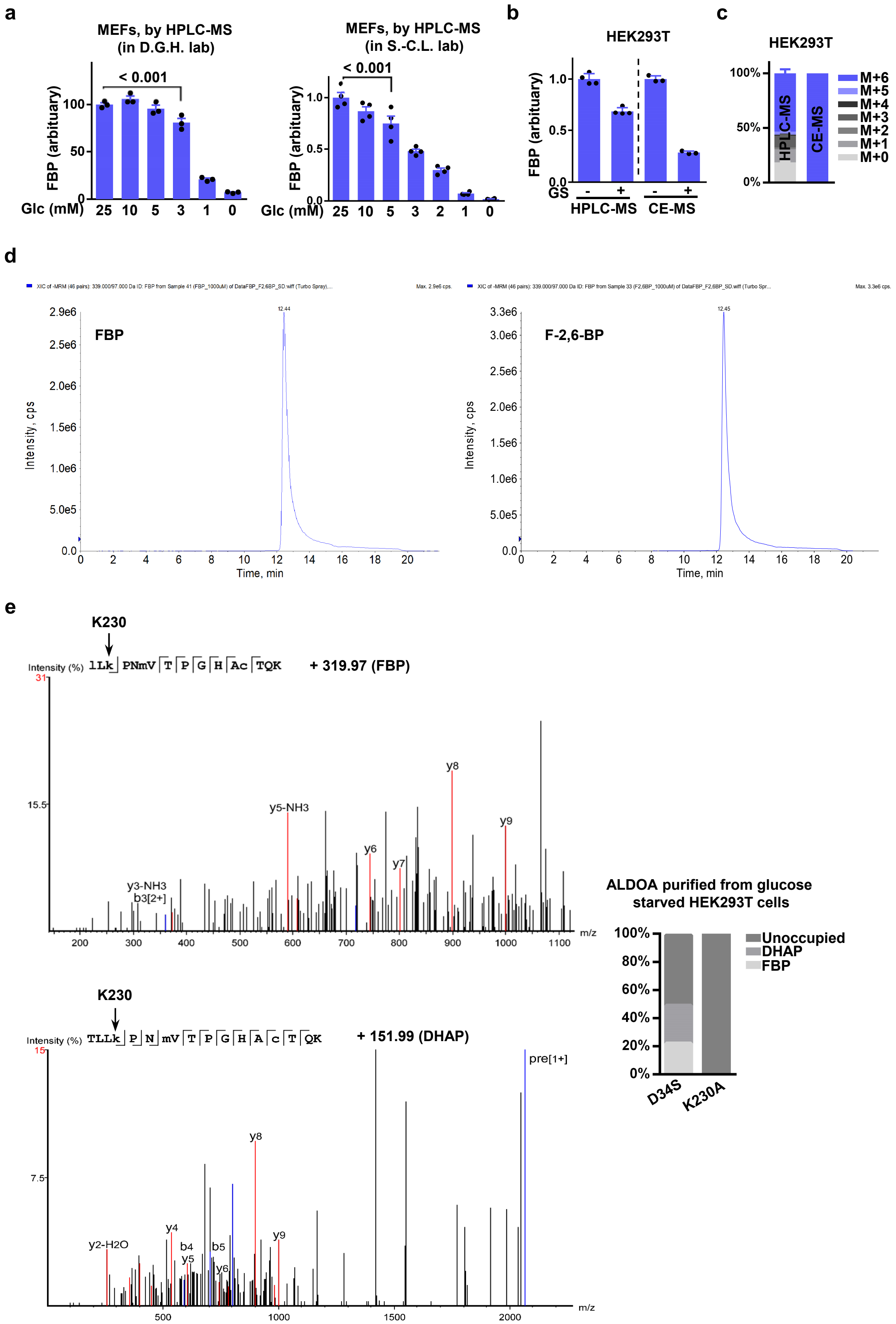

Supplementary information, Fig. S4 FBP is a critical metabolite that acts as an indicator of glycolytic rate.

a pHILIC columns can be applied to measure FBP levels in MEFs, but not in HEK293T cells. MEFs were incubated in DMEM medium containing glucose at the indicated concentrations for 2 h, followed by determining intracellular FBP levels via pHILIC column chromatography coupled with mass spectrometry. Independent results from the Hardie (left) and Lin (right) laboratories are shown.

b Glucose starvation leads to a large decrease of intracellular FBP levels in HEK293T cells measured by CE-MS. FBP levels were determined from HEK293T cells incubated in DMEM containing 25 mM glucose, or in glucose-free DMEM for 2 h. Values are presented as mean ± s.d., n = 3-4 for each treatment.

c Certain FBP isomers in HEK293T cells that behave similarly on the pHILIC column overlap the peak of FBP, because FBP should be entirely derived from glucose when glucose is the unique precursor for glycolysis. HEK293T cells were glucose starved for 2 h. The medium was then added with 25 mM [U-^{13}C]glucose. After another 15 min of incubation, the labelled and unlabelled FBP levels in cells were measured by LC-MS using a pHILIC column. Data were plotted as mean ± s.d., n = 4. Data from CE-MS were plotted from the results shown in a previously study[1].

d The pHILIC column fails to separate FBP from its isomer, fructose-2, 6-bisphosphate (F-2,6-BP). FBP and F-2,6-BP standards were subjected to chromatography on pHILIC column, followed by determining their MS/MS fragmentation patterns on a triple-Q mass spectrometer (SCIEX, QTRAP 5500). The spectrograms of an MS/MS fragment with m/z 97 of each isomer were shown, indicating the same retention time.

e The D34S mutant of aldolase is able to bind FBP as well as DHAP in low glucose. HEK-293T cells expressing HA-tagged ALDOA-D34S mutant, or ALDOA-K230A mutant as a control, were glucose starved for 2 h and lysed with buffer containing 200 mM $NaBH_4$ to trap Schiff-base intermediates. Aldolases were then immunoprecipitated. The potential phosphoglucitolylated (six-carbon) and a phosphoglycerolyated (three-carbon) K230 residue were determined by mass spectrometry. Typical spectrograms of phosphoglucitolylated and phosphoglycerolyated K230 residues (left), and their ratios on aldolase purified from starved HEK293T cells are shown (right).

Experiments in this figure were performed at least twice.

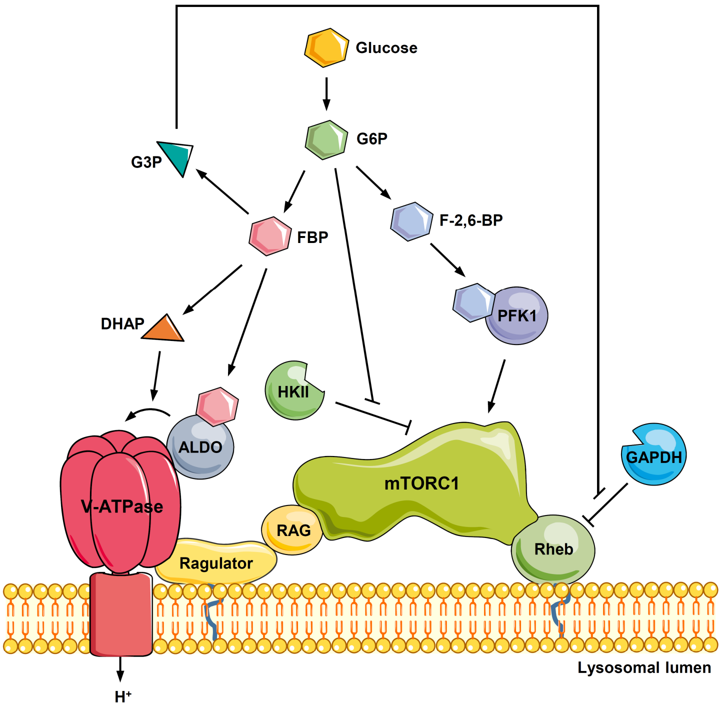

Supplementary information, Fig. S5 A simplified model depicting current understandings on the roles of glycolytic intermediary metabolites and their respective enzymes in controlling mTORC1 activity.

In high glucose, glucose-6-phosphate (G6P) converted by hexokinases (HKs), prevents HKII from binding and inhibiting mTORC1[2]. F-2,6-BP generated by phosphofructokinase 2 (PFK2), binds to and activates PFK1, promoting the lysosomal translocation of mTORC1[3]. FBP, perhaps also DHAP, binds to aldolase and maintains the activity of v-ATPase for mTORC1 activation. G3P binds GAPDH and prevents it from interfering with mTORC1-Rheb interaction[4]. These glucose metabolites and glycolytic enzymes thus regulate mTORC1 at multiple levels depending on glucose availability.

Methods

Plasmids. Point mutations of ALDOA and AXIN were performed by PCR-based site-directed mutagenesis using PrimeSTAR HS polymerase (Takara). Expression plasmids for various proteins were constructed in pBOBI vector for lentivirus packaging (stably expression). PCR products were verified by sequencing (Invitrogen, China). The lentivirus-based vector pLL3.7 was used for expression of siRNA in HEK293T cells and MEFs. The 19-nucleotide sequence for each siRNA is as follows: 5′-CACGAGTTGACCGGACATA-3′ for human AMPKα1, and 5′-GCCCAGATGAACGCTAAGA-3′ for human AMPKα2. The siRNAs against mouse AXIN were constructed and validated as described previously[5]. Sequences of all oligonucleotides used in this study are available upon request.

Reagents. Rabbit anti-phospho-S6K-T389 (cat. #9234, 1:1,000 for IB), anti-S6K (cat. #9202, 1:1,000 for IB), anti-AMPKα (cat. #2532, 1:1,000 for IB), anti-mTOR (cat. #2983, 1:100 For IF) and HRP-conjugated mouse anti-rabbit IgG (conformation specific, cat. #5127, 1:2,000 for IB) antibodies were purchased from Cell Signaling Technology. Rat anti-LAMP2 (cat. ab13524; 1:120 for IF) and mouse anti-LAMP1 (cat. ab25630, 1:120 for IF) antibodies were purchased from Abcam. Mouse anti-HA (cat. sc-7392, 1:2,000 for IB and 1:100 for IP) antibody was purchased from Santa Cruz Biotechnology. The HRP-conjugated goat anti-mouse IgG (cat. 115-035-003, 1:5,000 for IB) and HRP-conjugated goat anti-rabbit IgG (cat. 111-035-003, 1:5,000 for IB) antibodies were purchased from Jackson ImmunoResearch. Glucose (cat. G7021),

ConA (cat. C9705), capsaicin (cat. 360376), GSK-101 (cat. G0798), RN-1747 (cat. R1033), and $NaBH_4$ (cat. 71320) were purchased from Sigma. Hoechst (cat. 33342) and LysoSensor Green DND-189 (cat. L7535) were purchased from Molecular Probes. AMG-9810 (cat. sc-201477), BCTC (cat. sc-205599), FBP (cat. sc-214805), F-2,6-BP (cat. sc-491525) were purchased from Santa Cruz Biotechnology. [U-13C]glucose (cat. CLM-1396-PK) and [U-13C]glutamine (cat. CLM-1822) were purchased from Cambridge Isotope Laboratories. Protease inhibitor cocktail (cat. 04693116001) was purchased from Roche.

Cell culture and lentivirus infection. *AMPKα*$^{-/-}$ MEFs were kindly provided by Drs. Benoit Viollet and Keith R. Laderoute. HEK293T cells were obtained from ATCC (cat. CRL3216). HEK293T cells and MEFs were maintained in DMEM (Gibco, cat. 11965) supplemented with 10% fetal bovine serum (FBS), 100 IU penicillin and 100 mg/ml streptomycin at 37 °C in a humidified incubator containing 5% CO_2. Cells were verified to be free of mycoplasma contamination and authenticated by STR sequencing. Lentivirus for infection of the MEFs and HEK293T cells was packaged in HEK293T cells using Lipofectamine 2000 (Invitrogen, cat. 11668-027) transfection. At 30 h post transfection, medium was collected and added to the cells. The cells were incubated for another 24 h (for MEFs) or 12 h (for HEK293T cells). For starvation, cells were rinsed twice with PBS, and then incubated in glucose-free DMEM (Gibco, cat. 11966) supplemented with 10% FBS and 1 mM sodium pyruvate (Gibco, cat. 11360) for desired periods of time at 37 °C.

Immunoblotting. To analyse the levels of p-S6K, cells grown to 70-80% confluence in a well of a 6-well dish were lysed with 250 µl of ice cold lysis buffer. The lysates were then sonicated and centrifuged at 20,000 g for 10 min at 4 °C and an equal volume of 2X SDS sample buffer was added into the supernatant, and boiled for 10 min before immunoblotting. Levels of total proteins and phosphorylated proteins were analysed on separate gels, and representative immunoblots were shown. The band intensities on developed films were quantified using Image J software (National Institutes of Health Freeware).

Fluorescence microscopy. For determination the localisation of mTORC1, cells were grown on glass coverslips in 6-well dishes and were cultured to 60–80% confluence. Cells were fixed with 1 ml of 4% formaldehyde (diluted in PBS) at room temperature for 20 min. They were rinsed twice with 1 ml PBS (room temperature) and then permeabilised with 1 ml of 0.05% Triton X-100 (diluted in PBS) for 5 min at 4 °C. Cells were then rinsed twice with 1 ml of PBS, and were blocked in 1 ml of 5% normal goat serum (NGS, diluted in PBS), and then incubated with primary antibodies diluted in 5% NGS overnight at 4 °C. The cells were then rinsed three times with 1 ml of PBS, and then incubated with Alexa-Fluor 488-conjugated anti-rabbit secondary antibody (for staining mTOR, Molecular Probes, A21206; diluted 1:100 in 5% NGS) and Alexa-Fluor 594-conjugated anti-rat secondary antibody (for staining LAMP2, Molecular Probes, cat. A21209; diluted 1:100 in 5% NGS) or Alexa-Fluor 594-conjugated anti-

mouse secondary antibody (or staining LAMP1, Molecular Probes, cat. A11032; diluted 1:100 in 5% NGS) for 8 h at room temp at 4 °C. They were then washed four times with 1 ml of PBS, and then mounted with coverslip using ProLong Diamond Antifade Mountant (Molecular Probes, cat. P36970). Cells were imaged under a Zeiss LSM 780. Samples were excited with an Ar gas laser (Zeiss, laser module LGK 7812) using a 488-nm laser line for Alexa-Fluor 488 dye (green channel), and with a HeNe gas laser (Zeiss, LGK 7512 PF) using a 594-nm laser line for Alexa-Fluor 594 dye (red channel). Confocal microscope pictures were taken with a 63 × oil objective. The parameters, including 'PMT voltage', 'Offset', 'Pinhole' and 'Gain', were kept unchanged between each picture taken. For quantitative analyses of lysosomal mTOR localisation percentages (determined by Mander's overlap coefficient), the number of pixels from the red channel that overlap with pixels from the green channel are divided by the total number of pixels detected in the red channel above the threshold by ZEN 2010 software (Zeiss). Thresholds were set automatically by the software.

For detecting the pH of lysosomes, MEFs were grown on 35 mm of glass-bottom dishes, and were cultured to 60-80% confluence. Cells were treated with 1 μM (final concentration) LysoSensor Green DND-189 for 1 h, then washed twice with PBS and incubated in fresh, desired medium for another 30 min. In the meantime, 2 μg/mL (final concentration) Hoechst was added into the medium for staining nucleus before taking images. Hoechst were excited with a Diode laser using a 405-nm laser line, Lysosensor with an Ar gas laser (laser module LGK 7812) using a 488-nm laser line, and RFP with

a HeNe gas laser (Zeiss, LGK 7512 PF) using a 594-nm laser line. During imaging, live cells were kept at 37 °C, 5% CO_2 in a humidified incubation chamber (Zeiss, Incubator PM S1).

To determine the conformational changes of AXIN by FRET-FLIM, MEFs stably expressed with GFP, or GFP-AXIN (M2M4)-mCherry were grown on glass coverslips in 6-well dishes and were cultured to 60-80% confluence. Cells were then fixed with 1 ml of 4% formaldehyde (diluted in PBS) at room temperature for 20 min. Then the fluorescence lifetime of GFP (donor only) and GFP-AXIN (M2M4)-mCherry (donor with interactive proteins) was performed by Measuring Förster Resonance Energy Transfer Through Fluorescence Lifetime Imaging Microscopy (FRET/FLIM) using Leica SP8 Falcon. Data were analysed by LAS X Software (Leica).

Measurement of FBP by HPLC-MS. In the S-CL lab, to measure FBP levels in MEFs or HEK293T cells, one 10-cm dish (60-70% confluence) of cells were needed. Cells were rinsed with 20 ml PBS and instantly frozen in liquid nitrogen. Cells were then lysed with 1ml of methanol, and were scraped from the dish. The lysate was then mixed with 1ml of chloroform and 400 μl of water containing 20 μM [U-^{13}C]glutamine as an internal standard. The mixture was vortexed for 20 s. After centrifugation at 15,000 g for 15 min at 4 °C, 350 μl of aqueous phase was collected, lyophilized in a vacuum concentrator, and then dissolved in 100 μl of 50% acetonitrile (diluted in water). Measurement of FBP level was based on a QTRAP MS (SCIEX, QTRAP 5500)

interfaced with a UPLC system (Waters, ACQUITY UPLC system). Some 2 μl of each sample was loaded onto a SeQuant ZIC-pHILIC column (5 μm, 100 X 2.1 mm, Merck) equipped with a 5 mm pre-guard column to prevent from clogging, in a column oven at 40 °C. Mobile phase buffer A was 15 mM ammonium acetate in water (pH adjusted to 9.7 with ammonium hydroxide), and mobile phase buffer B 90% acetonitrile (in water). Before analysis, the column was equilibrated with 95% buffer B for 10 min at a flow rate 0.2 ml/min. The gradient was: 95% B for 2 min, then to 45% B within 13 min (linear gradient), then maintained for 3 min, then to 95% B directly, and maintained for 3 min. The flow rate was 0.2 ml/min. One blank was run between each sample to eliminate carry-over. The QTRAP mass spectrometer using an Turbo V ion source. The ion source was run in negative mode with a spray voltage of -4,500V, Gas1 40 psi, Gas2 50 psi and Curtain gas 35 psi. FBP was measured using the multiple reactions monitoring mode (MRM), with declustering potentials at -60 V and collision energies at -30 V (optimized using analytical standards of FBP). The following transition was used for monitoring FBP: 339/97. The relative amounts of FBP were analysed by MultiQuant Software (SCIEX).

In the DGH lab, FBP light and heavy form (Sigma) were used to optimize the LC-MS method for FSB detection and quantification. FBP levels were measured using a TSQ Quantiva mass spectrometer interfaced with an Ultimate 3000 Liquid Chromatography system (Thermo Scientific), equipped with a Sequant ZIC pHILIC column (50 x 2.1 mm, ID 5 μm; part no. 1.50459.0001; Merck). Mobile phase buffer A consisted of 0.3%

(v/v) formic acid adjusted to pH 9.15 with ammonia prior to a 1/10 dilution. Mobile phase buffer B was 50% methanol, 40% acetonitrile, 10% water (v/v). The column was maintained at a controlled temperature of 40 °C and was equilibrated with 60% buffer B for 6 min at a constant flow rate of 0.07 ml/min. Aliquots of 1 µl of each sample were loaded onto the column and compounds were eluted with a linear gradient of 60%-50% buffer B over 3 min, 50%-40% buffer B over 1 min, 40-15% buffer B over 5 min. The column was equilibrated again with 60% buffer B for 6 min. Eluents were sprayed into the TSQ Quantiva using an Ion Max NG ion source with the ion transfer tube temperature at 350 °C and vaporizer temperature at 30 °C. The TSQ Quantiva was run in negative mode with a spray voltage of 3,500, Sheath gas 40 and Aux gas 10 and sweep gas 2. Levels of FBP in samples were measured using MRM mode with optimized collision energies and radio-frequencies previously determined by infusing pure light and heavy FBP. One unique transition was used to monitor each of the compound, FBP (339/241), heavy FBP (345/246.98). Heavy FBP was used as an internal standard (40 µM final concentration) and was added to each sample after cell lysis in 5% PCA, prior to solvent neutralisation. One blank was run between each sample to eliminate carry-over.

Measurement of FBP by CE-MS. Sample preparation for CE-MS was carried out as described previously[6, 7]. Briefly, each measurement required cells to be collected from a 10-cm dish (60-70% confluence). Cells were rinsed with 20 ml of 5% mannitol solution (dissolved in water) and instantly frozen in liquid nitrogen. Cells were then

lysed with 1ml of methanol containing internal standards 1 [IS1 (Human Metabolome Technologies, H3304-1002, 1:200), used to standardize the metabolite intensity and to adjust the migration time], and were scraped from the dish. The lysate was then mixed with 1ml of chloroform and 400 μl of water by 20 s of vortexing. After centrifugation at 15,000 g for 15 min at 4 °C, 400 μl of aqueous phase was collected and was then filtrated through a 5 kDa cutoff filter (Millipore, cat. UFC3LCCNB-HMT) by centrifuging at 10,000 g for 3 h at 4 °C. The filtered aqueous phase was then lyophilized in a vacuum concentrator and then dissolved in 100 μl of water containing internal standards 3 [IS3 (Human Metabolome Technologies, H3304-1104, 1:200), to adjust the migration time]. Some 20 μl of redissolved solution was then loaded into an injection vial with a conical insert for CE-TOF MS (Agilent Technologies 7100, equipped with 6545 mass spectrometer) analysis. The relative amounts of FBP were analysed by MassHunter Quantitative Analysis Software (Agilent Technologies).

Determination of Schiff-base intermediate formation on K230 residue. For this experiment, each measurement (or condition) required aldolase to be purified from fifty 10-cm dishes (60–70% confluence) of HEK293T cells in which HA-tagged ALDOA-D34S or ALDOA-K230A (as a control) was stably expressed. Cells were lysed with 1 ml/dish of ice-cold lysis buffer containing 200 mM $NaBH_4$ (prepared by slowly adding $NaBH_4$ powder into a large volume, such as 200 ml of ice-cold lysis buffer with continuous stirring), followed by sonication and centrifugation at 4 °C for 15 min. Cell lysates were incubated with antibody against HA-tag for 4 h at 4 °C, and protein A/G

beads (1:250, balanced with lysis buffer containing $NaBH_4$) were then added into the lysate/antibody mixture for another 3 h at 4 °C. The beads were spun and washed with 100 times volume of lysis buffer for 3 times at 4 °C and then mixed with an equal volume of 2X SDS sample buffer for immunoblotting. After staining with staining solution (1% Coomassie Brilliant Blue R-250 dissolved in 45% methanol and 10% acetic acid in water) for 30 min, the SDS-PAGE gels were completely decoloured with staining solution without R-250 dye. The excised gel segments containing ALDOA band were subjected to in-gel trypsin digestion and dried. Samples were analysed on a nanoElute (Bruker) coupled to a timsTOF Pro (Bruker) equipped with a CaptiveSpray source. Peptides were dissolved in 10 μl 0.1% formic acid and were loaded onto a homemade C18 column (35 cm X 75 μm, ID 1.9 μm 100Å). Samples were then eluted for 60 min with linear gradients of 3-35% acetonitrile (in 0.1% formic acid) at a flow rate of 0.3 μl/min. Mass spectrum data were acquired with a timsTOF Pro mass spectrometer (Bruker) operated in PASEF mode. The raw files were analysed by Peaks Studio X software against uniprot database. According to the catalytic mechanisms reported[8], an adduct with 319.97 Dalton on K230 residue represented a phosphoglucitoylation (FBP) modification, or FBP binding, while 151.99 Dalton represented DHAP.

Statistical analysis. 1-way or 2-way ANOVA with post hoc analysis was used to compare values among different experimental groups. For experiments with only two groups, a two-tailed Student's t test was used as specified in the figure legends. For

ANOVA, the homogeneity of variance was tested by Levene's test. If the results are similar, the Tukey's test was proceeded, and if not, the Games-Howell's test was proceeded. Similar procedures were followed when Student's *t* test was performed. No samples or animals were excluded from the analysis. Tests were performed with Graphpad Prism 6, and $P < 0.05$ was considered statistically significant.

References for supplementary information

1. Zhang C. S. et al. *Nature* **548** 112-116 (2017).
2. Roberts D. J., Tan-Sah V. P., Ding E. Y., Smith J. M., Miyamoto S. *Mol Cell* **53** 521-533 (2014).
3. Almacellas E. et al. *iScience* **20** 434-448 (2019).
4. Lee M. N. et al. *Mol Cell Biol* **29** 3991-4001 (2009).
5. Zhang Y. L. et al. *Cell Metab* **18** 546-555 (2013).
6. Zhao J. et al. *Metabolomics* **10** 805-815 (2014).
7. Zhao Y. et al. *Sci Rep* **5** 16346 (2015).
8. Choi K. H., Shi J., Hopkins C. E., Tolan D. R., Allen K. N. *Biochemistry* **40** 13868-13875 (2001).
